# Knockout serotonin transporter in rats moderates outcome and stimulus generalization

**DOI:** 10.1038/s41398-020-01162-0

**Published:** 2021-01-07

**Authors:** Chao Ciu-Gwok Guo, Tao He, Joanes Grandjean, Judith Homberg

**Affiliations:** 1 Department of Cognitive Neuroscience, Donders Institute for Brain, Cognition, and Behaviour, Radboud University Medical Centre, Nijmegen, The Netherlands; 2 School of Psychological and Cognitive Sciences, Peking University, Beijing, China; 3 Department of Radiology and Nuclear Medicine, Radboud University Medical Centre, Nijmegen, The Netherlands

**Keywords:** Learning and memory, Psychiatric disorders, Predictive markers, Molecular neuroscience

## Abstract

Understanding the common dimension of mental disorders (such as anxiety, depression, and drug addiction) might contribute to the construction of biological frameworks (Research Domain Criteria, RDoC) for novel ways of treatment. One common dimension at the behavioral level observed across these disorders is a generalization. Testing generalization in serotonin transporter (5-HTT) knockout (KO) rats, an animal model showing depression/anxiety-like behaviors and drug addiction-like behaviors, could therefore provide more insights into this framework. We tested the outcome and stimulus generalization in wild-type (WT) and 5-HTT KO rats. Using a newly established touchscreen-based task, subjects directly responded to visual stimuli (Gabor patch images). We measured the response time and outcome in a precise manner. We found that 5-HTT KO rats processed visual information faster than WT rats during outcome generalization. Interestingly, during stimulus generalization, WT rats gradually responded faster to the stimuli as the sessions progressed, while 5-HTT KO rats responded faster than WT in the initial sessions and did not change significantly as the sessions progressed. This observation suggests that KO rats, compared to WT rats, may be less able to update changes in information. Taken together, KO 5-HTT modulates information processing when the environment changes.

## Introduction

Mental disorders, such as depression, anxiety, and drug addiction, are classified according to the symptoms described in the Diagnostic and Statistical Manual of Mental Disorders or International Statistical Classification of Diseases and Related Health Problems^1,2^. However, patients with one disorder tend to also have other disorders^3–5^. This comorbidity combined with the high burden and mortality of these disorders^6^ urged researchers to focus on the common dimensions of the disorders. This ultimately may foster the development of new treatments^7^. For this reason, a biological framework (Research Domain Criteria, RDoC) has been constructed that aims to understand dimensions that cut across psychiatric disorders and are shaped by a specific constellation of molecules, cells, and behaviors^8^. The biological framework can be the basis for objective measurements of psychopathology and can provide leads for personalized treatments^9^.

One common dimension of the biological framework observed across anxiety, depression, and drug addiction in both humans and animals is generalization^10–15^. Generalization has recently been proposed as one dimension in the RDoC framework^16^. The processing of familiar stimuli with less predictable outcomes was termed as outcome generalization, while stimulus generalization refers to the ability to transfer the learned information predicted by stimuli to novel information predicted by other stimuli. Generalization reduces the effort for individuals to learn the prediction of the outcome of each stimulus from scratch, but can use it to guide behavior. However, too broad or too narrow generalization may lead to maladaptation, as this may cause approaching stimuli indiscriminately^17,18^, avoiding stimuli^13,19^, or nonresponding to stimuli^15,20^. This approach is unlikely to allow individuals to optimally or efficiently apply the stimuli–outcome association obtained for specific stimuli to other related stimuli, resulting in the occurrence of a disorder^18^.

In stimulus generalization, the relationship between conditioned stimuli (CS) and generalization stimuli (GS) is determined by the similarity of stimuli at one or more perceptual dimensions (e.g., frequency of sound, frequency of object vibration, tactile feel of the material, or angle of grating)^15^. The response choice to GS is typically more robust as its similarity is closer to the CS^21^. Before making choices, it requires subjects’ response time (RT) to process the external and internal information of stimuli during both stimulus and outcome generalization. RT was initially proposed as a key readout by Donders^22^ and is considered to play a crucial role in information processing both in humans and animals^23,24^. Early research in humans found that stress shortened RT, anxious personalities had faster RT, and drug users reduced RT to drug-related signals^25^. Moreover, serotonin transporter (5-HTT) inhibition shortened RT without altering discrimination in healthy humans^26,27^ but impaired generalization in depressive patients^11^. Besides, the lower expression of 5-HTT in humans is associated with higher generalized anxiety and depression^28,29^. Rodent studies have demonstrated that 5-HTT-knockout (KO) rodents show increased anxiety- and depression-like behavior^30,31^, whereas they also self-administer higher amounts of cocaine^32,33^. However, no impairment of visual discrimination was observed^34^. Accordingly, it has been suggested that 5-HTT-related disorders may be caused by maladaptive information processing^17,35^. The (mal)adaptation in 5-HTT KO animals can be directly measured by testing their stimulus generalization ability. In a simple visual generalization task, 5-HTT KO mice exhibited a similar generalization curve compared to wild-type (WT) control mice^36^. However, only one type of generalization stimulus was presented and the RT to the stimuli was not reported in this experiment. In another experiment, in which space (arm) generalization was measured, 5-HTT KO mice showed a tendency toward an increase in RT to the generalization arm^37^. However, only the RT was measured and the generalization curve was not reported in this task. Other serotonin-relevant studies showed that serotonergic neurons are encoding reward-related information processing. Both expected and unexpected rewards activate serotonergic neurons and the serotonergic neurons fired tonically when the animal was waiting for the reward^38^. In addition, optogenetically activating the serotonergic neurons promotes waiting for duration^39^. Also, our previous study showed that 5-HTT KO rats spent more time exploring the CS, especially when there was no expected reward outcome^40^. These findings indicate that serotonin affects RT in rats during information processing.

In the current study, we tested the effect of knocking out 5-HTT in rats on outcome and stimulus generalization performance. By testing generalization in 5-HTT KO rats, an animal model showing depression/anxiety- and drug addiction-like behaviors^17,30,31^, we could provide more insights into constructing generalization as one of the dimensions in the biological framework of RDoc. The visual CS and GS used in the experiment were presented on a touchscreen so that subjects could directly respond to the stimuli and the RT could be measured in a precise manner.

## Materials and methods

### Subjects

Twenty male rats weighing 325–400 g and aged 80–100 days were served as subjects (10 5-HTT KO and 10 WT rats). Sample sizes were based on our previous studies^34,41^. Both investigators and caregivers were blinded to the groups during experiments. The KO rats (Slc6a41Hubr) have been generated by target-selected ENU-induced mutagenesis and had been outcrossed for at least 15 generations with commercial Wistar rats^42^. All rats were housed by pairs of the same group in a temperature-controlled room (21 ± 1 °C) with 40–50% humidity under a 12/12-h reversed light–dark cycle (bright light at 19:00 to 7:00) at regular Eurostandard type III H cages including shelter. Rats had ad libitum access to water and chow in their home cages. Experiments were approved by the Animal Welfare Committee of Radboud University Medical Center, Nijmegen, the Netherlands. Rats were sacrificed at the end of the study and their brains were preserved for further analysis.

### Behavioral procedures

#### Apparatus

**Fig. 1:**
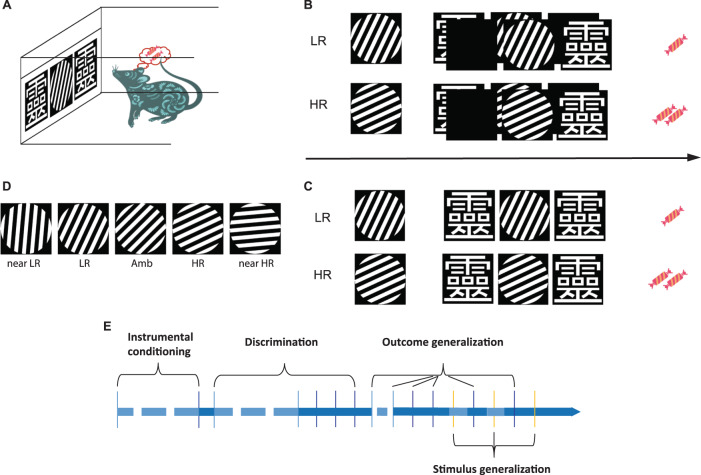
A representative schematic view of the experimental procedures. **A** A representative rat in an operant chamber equipped with a touchscreen. **B** A high-reward (HR) trial and a low-reward (LR) trial sequence in the stage of instrumental learning. The rat was allowed to touch the stimulus of white and black gratings (phase one), which was followed by the random presentation of a reporter image “靈” on either the left or the right side of the screen (phase two). Then, the rat was allowed to touch this single reporter image, leading to the delivery of one sucrose pellet for LR, and two sucrose pellets for HR trials (phase three). **C** A HR and an LR trial sequence in the stage of discrimination learning. During a HR trial, the rat was allowed to touch the 65° grating stimulus (phase one). Then, if it was touched, two reporter images were presented on the left and right side of the screen simultaneously (phase two). A correct response (reporter image on the right screen was touched) produced two sucrose pellets (phase three). During an LR trial, the rat was allowed to touch the 25° grating stimulus (phase one). Then if it was touched, two reporter images were presented on the left and right side of the screen simultaneously (phase two). A correct response (reporter image on the left screen was touched) produced one sucrose pellet (phase three). An incorrect response initiated a correction trial for both LR and HR trials. Correction trial is the same type of trial as the previous trial during which the subject responded incorrectly. **D** A representation of all the stimulus images during the generalization phase. The degrees of LR and HR stimuli are 25° and 65°, respectively. The near-LR stimulus is 5°, the near-HR stimulus is 85°, and the ambiguous (Amb) stimulus is 45°. **E** The experiment flowchart. Each vertical line represents one session and the horizontal line represents the timeline. The dash horizontal lines denoting the sessions between the two vertical lines are the same. Solid lines between two vertical lines denoting the two sessions represented by the lines took place on two consecutive days. Blue lines represent sessions during instrumental conditioning and discrimination. Light-blue vertical lines represent the sessions that the ratio of correct response is below the criteria (90% correct trials per session for instrumental conditioning, 70% correct trials per session for discrimination). Dark-blue vertical lines represent the sessions that rats reached the criteria. Yellow vertical lines represent sessions during stimulus generalization.

The behavioral training and tests were performed in eight computer-controlled operant chambers (Med Associates). Each chamber was equipped with a houselight for illuminating the chamber, a touchscreen for presenting stimuli, a metal panel for dividing the screen into three windows, and a dispenser for delivering sucrose pellets (TestDiet, St. Louis, USA). Each chamber was cleaned right at the start and after the experiment every day. The experimental procedures and data acquisition were programmed in K-limbic software (Med Associates, Hertfordshire, UK). Images presented on the screen were all generated by using Python (version 3.7) with the package of PsychoPy (version 3.0). A schematic view of a rat in a chamber is presented in Fig. 1A.

#### Training and test

All rats were handled for 3 days before training in the touchscreen box. Rats were trained and tested every day. The behavioral procedure was modified from a previous touchscreen task developed by us^41^. Briefly, animals were trained well to associate touching stimuli with acquiring rewards in the stage of instrumental conditioning. In the next stage, the animals were trained to discriminate visual stimuli predicting lower reward (LR) and higher reward (HR). Instrumental conditioning took place when only one reporter stimulus (靈) was present, whereas discrimination took place when two reporter stimuli (靈) were present at the same time. Once rats were trained well in visual discrimination, they were tested in an additional visual discrimination stage during which the expected reward outcome was reduced, based on a probability of 75% instead of 100% to test outcome generalization. Finally, rats were tested for stimulus generalization. The flowchart of the training and testing is shown in Fig. 1E. The procedures of each stage are thoroughly described in the Supplementary Methods section. All stimuli used in the study can be downloaded from the Donders Institute for Brain, Cognition, and Behavior Repository at http://hdl.handle.net/11633/aadis56o.

### Data analysis

Data were analyzed using t tests in software JASP and mixed-effect linear models in R software (version 3.6) with package brms^43^. Using mixed-effect models to analyze the data reduces the possibility of false positives^5^. The numbers of sessions to reach the learning criterion, learning rate (the slope for the animals reaching the learning criterion) were analyzed using a Bayesian *t* test. If the normality assumption was violated before performing the *t* test, a Bayesian Mann–Whitney *U* test was applied. Data were plotted with the library DABEST^44^ in Python (version 3.7). They statically examined by estimating a Bayes factor^45^ by comparing the fit of the data under the null and the alternative hypothesis. The Bayes factor provides the information: which hypothesis is favored by the given data. To obtain the parameters for the generalization curve for each rat, data (proportion toward HR response of each rat) were fitted to the psychometric function using the Palamedes Matlab toolbox^46^. The psychometric function is given by

$$\psi \left( {{\mathrm\it{x}};\alpha ,\beta ,\lambda } \right) = \lambda + \left( {1 - 2\lambda } \right){\mathrm{F}}\left( {{\mathrm\it{x}};\alpha ,\beta } \right)$$

where *ψ* represents the proportion toward HR responses, *α* and *β* are two free parameters that denote the location (threshold) and slope of the psychometric function separately, and *λ* accounts for stimulus-independent lapses and was fixed to 0.01. *x* denotes the grating orientation difference between left and right stimuli. F is a cumulative Gaussian distribution that is given by

$${\mathrm{F}}\left( {{\mathrm\it{x}};\alpha ,\beta } \right) = \frac{{\beta }}{{\sqrt {2\pi } }}{\int}_{\! - \infty }^{\mathrm\it{x}} {\rm{exp}\left( { - \frac{{{\beta}^2\left( {{\it{x}} - \alpha } \right)^2}}{2}} \right)}$$

Crucially, *α* corresponds to the point of subjective equality (PSE). The PSE was defined as the midpoint of the psychometric function, at which the stimulus was perceived equally often as tilted to the right and to the left. Discrimination accuracy was defined as the percentage of correct responses during the stage of discrimination (correction trials were not included). In stimulus generalization, the percentage of correct responses to LR and HR stimulus was defined as generalization accuracy. The percentage of incorrect responses to near-LR and near-HR stimuli was defined as generalization error. The percentage of interpreting the ambiguous stimulus as LR or HR in ambiguous trials was defined as generalization bias. Data were then statistically analyzed using the Bayesian mixed-effect linear model with the brms package. This model provides multiple distribution families, including Gaussian, Beta, and shifted log-normal distributions for our data fitting (see the distribution of model fitted and observed values in Supplementary Fig. 1). In these tests, genotype, session, and stimuli factors were entered as fixed effects; subject and date of the experiment were entered as random effects; the session was also entered as a random slope, unless stated otherwise. The priors were set as default and their influence on the results will be negligible^43^. The contrast was set to sum-to-zero (deviation coding). Significant effects were calculated by 95% credible intervals (CrI) and the estimate (*E*) of the effect was given. If the 95% CrI did not include 0, the effect was deemed “significant”.

Three KO and two WT rats were excluded from the mixed-effect analysis for the discrimination stage since they did not reach the discrimination criterion after 45 days of training (at least 70% correct responses for 3 consecutive days). Considering that readers in the field of biological neuroscience might not be familiar with Bayesian mixed-effect models, we provide the maximal statistical information by producing robust and transparent data illustration^47^ for probability density (violin plot), individual observations and outliers (dot plot), and mean, median, and quantile of data (box plot), unless stated otherwise. All data and code are available from the Donders Institute for Brain, Cognition, and Behavior Repository at http://hdl.handle.net/11633/aadis56o.

## Results

### KO and WT rats might acquire the instrumental task at a similar speed

**Fig. 2:**
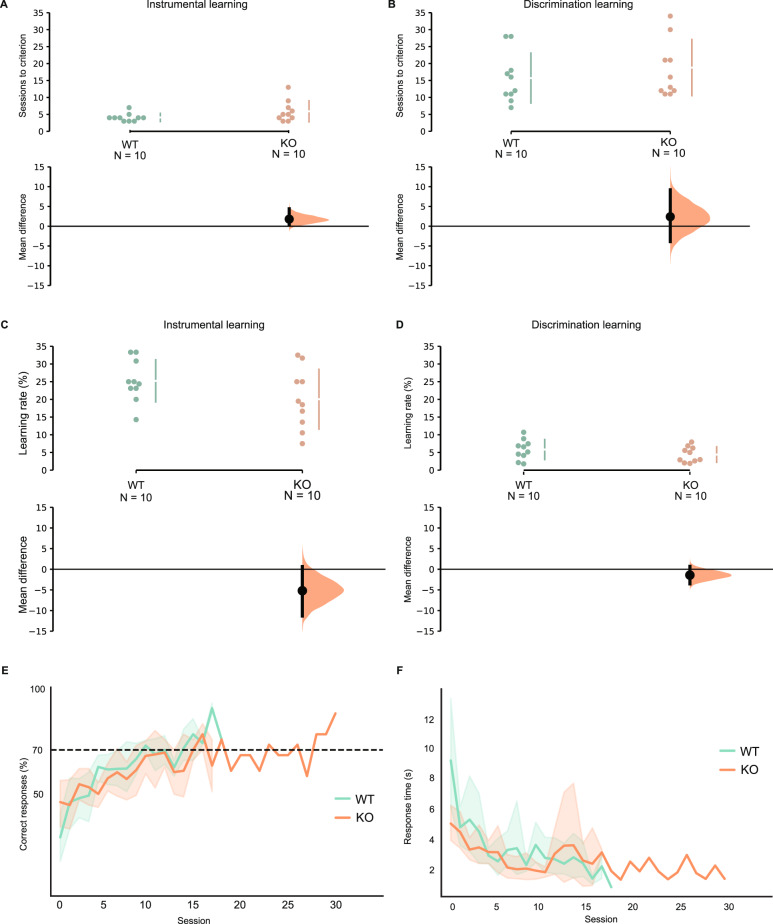
Instrumental and discrimination learning: all sessions. **A**–**D** Individual data are shown as dots. The effect size and 95% confidence intervals obtained from bootstrapping are plotted on separate axes beneath the individual data points. For each genotype, mean ± standard deviations are shown as vertical gapped lines. **A**, **B** The number of sessions. **A** Instrumental learning. The number of sessions the rats needed to reach the learning criterion. There was no difference in the number of sessions needed between WT and KO rats. **B** Discrimination learning. The number of sessions the rats needed to reach the learning criterion or quit. There was no difference in the number of sessions needed between them. **C**, **D** Learning rate. **C** Instrumental learning. The instrumental learning rate was not significantly different between KO and WT rats. **D** Discrimination learning. The discrimination learning rate was not significantly different between KO and WT rats. **E** Discrimination accuracy. Mean percentage with 95% confidence intervals of correct responses across all learning sessions. The stimulus discrimination accuracy increased significantly as learning progressed in both WT and KO. There was no significant difference in correct responses across all learning sessions between KO and WT rats. **F** Response time during discrimination. Mean with 95% confidence intervals of response time across all learning sessions. The response time decreased significantly as learning progressed in both WT and KO rats. Genotypes were not significantly different. **E**, **F** WT (*N* = 8), KO (*N* = 7).

We trained rats to learn to associate touching sequential stimuli with acquiring rewards. All rats reached the learning criterion (≥90% correct trials in the last session). To assess whether both KO and WT rats can learn the task at the same speed overall, the total number of sessions needed to reach the criterion was compared between the two genotypes (see Fig. 2A). An estimated Bayes factor from the Bayesian Mann–Whitney *U* test (BF_01_ = 1.488) suggested that it was 1.488 times more likely there was no genotype difference than there was genotype difference. In addition, the learning rate between the KO and WT rats was plotted in Fig. 2C. An estimated Bayes factor from the Bayesian *t* test (BF_01_ = 1.067) suggested that it was 1.067 times more likely there was no genotype difference of learning rate than there was genotype difference (see Fig. 2C). Both Bayes factors were below 3, indicating that the evidence for supporting the hypothesis of no genotype difference was weak. Therefore, we concluded that rats from both genotypes might learn the instrumental task at a similar speed.

### The discrimination performance might be similar between WT and KO rats

Next, we trained rats to perform a visual discrimination task categorizing Gabor patches as either HR or LR stimulus. The number of training sessions needed to reach the learning criterion of at least 70% accuracy for 3 consecutive days or on the 45th day (when all rats were sacrificed) was analyzed. The number of sessions needed is presented in Fig. 2B. An estimated Bayes factor (BF_01_ = 1.691) suggested that it was 1.691 times more likely there was no genotype difference than there was genotype difference. In addition, a Bayesian *t* test for the learning rate revealed that the estimated Bayes factor was BF_01_ = 1.463. The factor suggests that it was 1.463 times more likely there was no genotype difference than there was a genotype difference (see Fig. 2D). Both Bayes factors were below 3, indicating that the evidence supporting the hypothesis of no genotype difference was weak. Therefore, we concluded that both KO and WT rats could learn to discriminate the LR and HR stimuli, possibly at a similar speed.

The training session’s effect on discrimination accuracy and RT was further analyzed. As shown in Fig. 2E, as the sessions proceeded, the discrimination accuracy increased significantly (*E* = 0.05, CrI = [0.03, 0.09]). This test was not significantly different between KO and WT rats (*E* = −0.17, CrI = [−0.44, 0.1]), and no significant genotype*session interaction effect was found (*E* = 0.01, CrI = [−0.04, 0.06]). This indicates that both KO and WT rats learned to discriminate the stimuli with similar accuracy.

The average RT of each session for touching the stimuli across the whole training session was also analyzed, which is presented in Fig. 2F. As the session progressed, the RT decreased significantly (*E* = −0.09, CrI = [−0.13, −0.06]). No other significant differences were found in this test (genotype: *E* = −0.22, CrI = [−0.66, 0.20]; genotype*sessions: *E* = 0.01, CrI = [−0.06, 0.07]). This indicates that KO and WT rats processed the signal of CS similarly.

Taking the results above, we conclude that KO and WT rats might acquire the ability to discriminate the LR and HR stimuli similarly.

### Less reward-predictable context altered RT

**Fig. 3:**
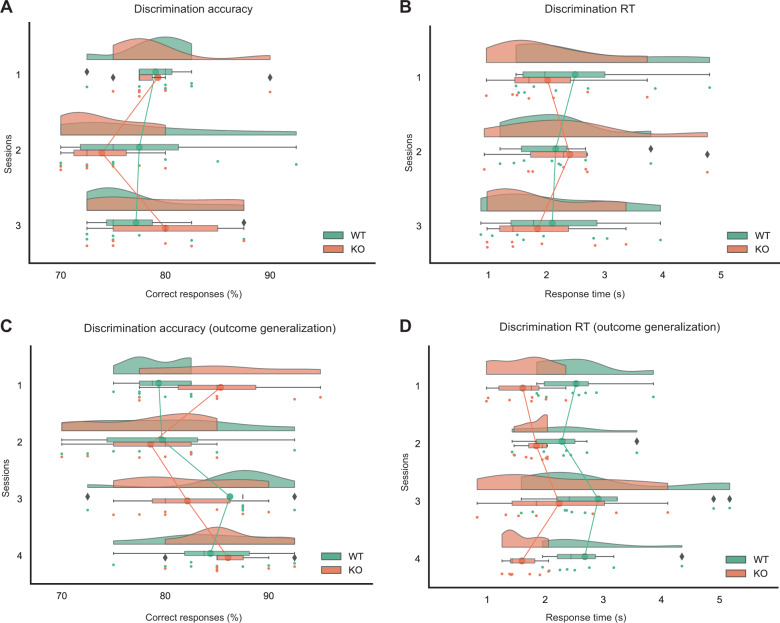
Discrimination with and without the change in reward contingencies (accuracy ≥ 70%). **A**, **B** Discrimination performance without a change in reward contingencies. **A** Discrimination accuracy. Percentage of correct responses across the last three sessions. The discrimination accuracy stayed similar as the session progressed in both WT and KO rats. There was no significant difference in correct responses across the last three sessions between them. **B** Discrimination RT. The response time stayed similar as the session progressed in both KO and WT rats. There was no significant difference in RT across the sessions between them. **C**, **D** Discrimination performance when probabilistic reward contingencies were reduced (outcome generalization). **C** Discrimination accuracy. Percentage of correct responses across sessions. No significant difference between KO and WT rats, and no significant difference across sessions. **D** Discrimination RT. The RT was significantly lower in KO than WT rats. There was no significant effect of the sessions. Note: WT (*N* = 7); KO (*N* = 8); the points between solid lines represent the mean of the group; the dots represent individual data; the hills represent the probability distribution of the individual data; the range of the colored box represents the interquartile range; the vertical line in the colored box represents the group median; the range of colored box with whiskers on both sides represents the minimum and maximum data range; data outside the whiskers are outliers denoted by the symbol ⧫.

The above results are consistent with previous findings that there is no impairment in visual discrimination in 5-HTT KO versus WT rats if individuals are in a stable environment^34^. When the environment changes, maladaptive behavior may become overt in 5-HTT KO rats^35^. To test this, we analyzed the discrimination accuracy and RT when the animals reached the learning criterion with and without the change in reward contingencies. As shown in Fig. 3A, when the reward contingencies were not changed, there were no significant effects on discrimination accuracy in this test (genotype: *E* = –0.02, CrI = [–0.24, 0.28]; session: *E* = −0.01, CrI = [−0.16, 0.13]; genotype*session: *E* = −0.08, CrI = [−0.19, 0.36]). Figure 3B shows that there were also no significant effects on RT (genotype: *E* = −0.08, CrI = [−0.84, 0.65]; session: *E* = −0.16, CrI = [−0.49, 0.12]; genotype*session: *E* = 0.08, CrI = [−0.52, 0.69]). The results indicate that both WT and KO rats had a similar discrimination performance when reward contingencies were not changed.

The discrimination accuracy was also not significantly different between KO and WT rats when probabilistic reward contingencies were reduced (*E* = 0.08, CrI = [−0.19, 0.36]; session: *E* = 0.09, CrI = [−0.01, 0.20]; genotype*session: *E* = −0.14, CrI = [−0.34, 0.08], see Fig. 3C). However, the RT was significantly lower in KO rats than in WT rats (*E* = −0.47, CrI = [−0.82, −0.13], see Fig. 3D). No other significant effects were observed (session: *E* = 0.03, CrI = [−0.07, 0.14]; genotype*sessions: *E* = −0.03, CrI = [−0.22, 0.16]). The data indicate that KO rats responded to the stimuli faster than WT rats when the reward outcome was less predictable.

### Generalization accuracy, error, bias, and generalization curve are similar between KO and WT rats

**Fig. 4:**
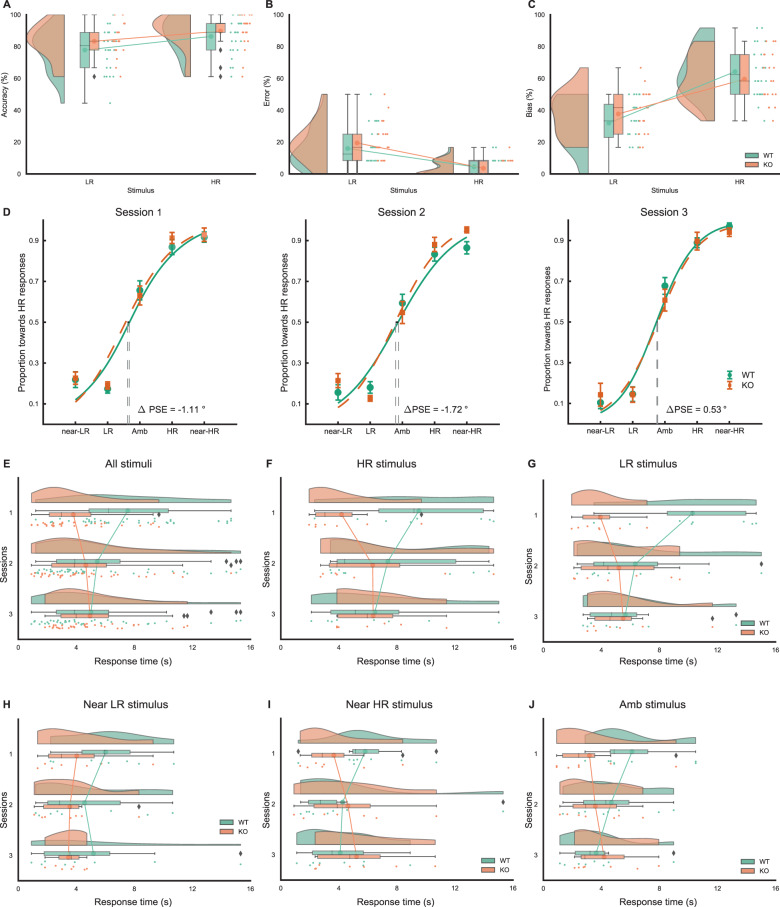
Stimulus generalization. **A** The generalization accuracy. Percentage of correct responses across sessions to LR and LR stimuli. Rats showed a significantly higher generalization accuracy to the HR-conditioned stimulus than to the LR-conditioned stimulus in both WT and KO rats, but no significant genotype effect. **B** Generalization error. The percentage of incorrect responses to near-LR and near-HR stimuli. Rats made significantly fewer generalization errors in response to the near-HR generalization stimulus than the near-LR generalization stimulus but not genotype effects. **C** Generalization bias. Percentage of responses to interpret the ambiguous stimulus as LR or HR. Rats displayed a significantly higher generalization bias to HR in response to ambiguous stimuli, but there was no significant genotype effect. **D** Generalization curve. WT and KO rats had similar generalization curves. Error bars: mean with 95% confidence intervals. ΔPSE = KO − WT. **E**–**J** Response time (RT) during generalization. **E** Interaction (session and genotype) effect on RT. The response time between the genotype of KO and WT rats to each session across all stimuli. There was a significant interaction difference between genotype and session across all stimuli. **F** RT to LR stimulus. There was a significant genotype*session interaction for the RT assessed across three sessions. The main effect of genotype or session was not significant. **G** RT to HR stimulus. The RT in KO and WT rats across three sessions. There was a significant interaction effect between genotype and session. The main effect of genotype or session was not significant. **H** RT to near-LR stimulus. The RT between KO and WT rats across three sessions. There was no significant interaction effect between genotype and session. The main effect of genotype or session was not significant. **I** RT to near-HR stimulus. The RT between KO and WT rats across three sessions. There was no significant interaction effect between genotype and session. The main effect of genotype or session was not significant. **J** RT to ambiguous stimulus. The RT between KO and WT rats across three sessions. There was a significant interaction effect between genotype and session. The main effect of genotype or session was not significant. Note: KO (*N* = 7); WT (*N* = 8). The point between solid lines is the mean of the group; the dots represent individual data; the hills represent the probability distribution of the individual data; the range of the colored box represents the interquartile range; the vertical line in the colored box represents the group median; the range of colored box with whiskers on both sides represents the minimum and maximum data range; data outside the whiskers are outliers denoted by the symbol ⧫.

The generalization accuracy measured across three sessions for both KO and WT rats is presented in Fig. 4A. We found that rats displayed a significantly higher generalization accuracy to the HR-conditioned stimulus than the LR-conditioned stimulus (*E* = 0.18, CrI = [0.05, 0.32]). No other significant effects were observed in this test (genotype: *E* = 0.23, CrI = [−0.27, 0.71]; session: *E* = 0.11, CrI = [−0.18, 0.40]; genotype*session: *E* = −0.07, CrI = [−0.66, 0.5]; genotype*stimulus: *E* = 0.06, CrI = [−0.35, 0.22]). The generalization error measured across three sessions for both KO and WT rats is presented in Fig. 4B. The data show that rats made less generalization errors in response to the near-HR generalization stimulus than to the near-LR generalization stimulus (*E* = −0.40, CrI = [−0.59, −0.21]). Other effects were not significantly different (genotype: *E* = 0.16, CrI = [−0.27, 0.57]; session: *E* = −0.15, CrI = [−0.44, 0.13]; genotype*sessions: *E* = 0.14, CrI = [−0.40, 0.67]; genotype*stimulus: *E* = 0.01, CrI = [−0.37, 0.36]). The generalization bias across three sessions for both KO and WT rats is presented in Fig. 4C. We found that rats had a higher generalization bias to HR than LR in response to ambiguous stimuli (*E* = 0.53, CrI = [0.36, 0.66]). No other effects were significantly different (genotype: *E* = −0.06, CrI = [−0.34, 0.22]; session: *E* = 0.01, CrI = [−0.17, 0.21]; genotype*sessions: *E* = −0.07, CrI = [−0.43, 0.3]; genotype*stimulus: *E* = −0.17, CrI = [−0.43, 0.30]). Figure 4D shows that KO and WT rats have similar generalization curves. Both genotypes responded to the GS in a more robust manner as its similarity was closer to CS. The locations (PSE) of the curves between WT and KO rats were similar (genotype: *E* = −0.5, CrI = [−0.63, 5.06]; session: *E* = 0.64, CrI = [−2.76, 4.01]; genotype*session: *E* = 1.4, CrI = [−4.86, 7.54]) and the slopes of the curves between WT and KO rats were also similar (genotype: *E* = −0.04, CrI = [−0.57, 0.49]; session: *E* = 0.24, CrI = [0.00, 0.47]; genotype*session: *E* = −0.04, CrI = [−0.49, 0.42]).

The results indicate that generalization accuracy, error, and bias were similar in KO and WT rats, and that both KO and WT displayed a bias to HR when responding to ambiguous stimuli. Also, generalization curves were similar between KO and WT rats, and as the similarity was closer to CS, the responses to GS were more robust.

### RT tends to converge between KO and WT rats during stimulus generalization

RT is considered to play a crucial role in information processing both in humans and animals^23,24^. To examine the effect of genotype on information processing during generalization, RT was analyzed. There was no significant effect of genotype over the three sessions in all stimulus conditions on RT as shown in Supplementary Fig. 2A (*E* = −0.28, CrI = [−0.92, 0.36]), and no significant effect of session on RT overall (Supplementary Fig. 2B, E = −0.04, CrI = [−0.22, 0.13]). However, every stimulus had a significant effect on RT as shown in Supplementary Fig. 2C (near-LR: *E* = 1.17, CrI = [0.81, 1.51]; LR: *E* = 0.23, CrI = [0.11, 0.36]; ambiguous: *E* = −0.21, CrI = [−0.33, −0.10]; HR: *E* = 0.31, CrI = [0.18, 0.43]; near-HR: *E* = −0.14, CrI = [−0.26, −0.02]). As shown in Fig. 4E, there was a significant interaction effect between genotype and session across the stimuli (*E* = 0.46, CrI = [0.13, 0.83]). The follow-up analysis showed that there was a significant effect of session in WT rats. RT decreased in WT rats as sessions increased (*E* = −0.36, Crl = [−0.66, −0.06]). In 5-HTT KO rats, we only observed an increased tendency (not significantly, see Fig. 4E) for an increase in RT (*E* = 0.22, Crl = [−0.14, 0.56]). To further examine the interaction effect between genotype and session on RT under each stimulus, analysis of RT function on each stimulus was modeled.

For the LR stimulus (Fig. 4F), there was a significant interaction effect between genotype and session (*E* = 0.76, CrI = [0.15, 1.47]). The main effects of genotype and session were not significant when the LR stimulus was presented (genotype: −0.59, CrI = [−1.45, 0.26]; session: *E* = −0.07, CrI = [−0.39, 0.27]). For the HR stimulus (Fig. 4G), there was a significant interaction effect between genotype and session (*E* = 0.79, CrI = [0.18, 1.48]). However, genotype and session effects were not significant under the LR condition (genotype: −0.45, CrI = [−1.30, 0.41]; session: *E* = 0.08, CrI = [−0.24, 0.41]). For the near-LR stimulus (Fig. 4H), no significant effects were found (genotype: *E* = −0.26, CrI = [−1.2, 0.69]; session: *E* = −0.17, CrI = [−0.49, 0.13]; genotype*session: *E* = 0.31, CrI = [–0.27, 0.93]). For the near-HR stimulus (Fig. 4I), there were no significant effects (genotype: *E* = −0.07, CrI = [−0.75, 0.66]; session: *E* = 0.01, CrI = [−0.30, 0.36]; genotype*session: *E* = 0.47, CrI = [−0.14, 1.09]). For the ambiguous stimulus (Fig. 4J), there was a significant interaction effect between genotype and session (*E* = 0.74, CrI = [0.09, 1.49]). No other significant effects were found (genotype: *E* = −0.47, CrI = [−1.15, 0.29]; session: *E* = 0.00, CrI = [−0.33, 0.36]). We concluded that when processing the information from the HR and LR CS and ambiguous generalization stimulus during generalization, KO rats may have a faster RT than WT rats at the initial sessions.

## Discussion

We presented Gabor patches to rats in our newly developed touchscreen-based task. The Gabor patches are widely used visual stimuli in human experiments^48^. Combining touchscreen and Gabor patch stimuli in the same experiment greatly increases the translational value of the animal data to humans^49^. Our study found that 5-HTT KO rats responded faster to the stimulus than WT rats during outcome generalization where reward contingencies were probabilistically reduced. After responding to the GS, WT rats appeased to increase response speed to the stimuli as the sessions progressed, while 5-HTT KO rats responded faster than WT rats in the initial sessions. KO rats did not change response speed significantly, but might tend to decrease during the stage of stimulus generalization. Notably, no significant differences in response accuracy and the generalization curves were observed between KO and WT rats.

The present findings are similar to the previous finding that there is no difference in reward instrumental conditioning and discrimination between KO and WT rats^34^. The present results show that BFs during instrumental conditioning and discrimination were slightly more than 1. This indicates that the evidence to support the null hypothesis (WT = KO) is according to Jeffreys’ classification (1961) weak. A recent paper also indicated that the BF01 should be at least more than 3 to have moderate evidence to support the null hypothesis^50^. Indeed, Bayesian t testing provides increasing evidence for the absence of an effect (effect size equals to 0) with increasing sample size. However, evidence for the null hypothesis becomes substantially harder to provide and requires larger sample sizes when applying a two-tailed *t* test^50^. We used a two-tailed *t* test based on the null hypothesis that KO rats could perform similarly as WT rats in our discrimination task: a previous study also did not observe group differences in a different version of discrimination learning but a significant effect in reversal learning^51^. Increasing sample size to at least 100 might be needed when expecting that the effect size equals 0^50^. However, 100 rats per group as a sample size might not be practical in one experiment when considering animal welfare and ethics. Rather, considering the large individual differences in generalization in humans^52^, it might be beneficial to understand what other possible molecular mechanisms drive the individual differences in WT rats in a big sample size.

Fast information processing is crucial for organisms to adapt to changes in the external environment^53^. 5-HTT KO rats were faster in information processing than WT rats under the less predictable context of uncertainty. Serotonin may play a critical role of tracking uncertainty in both rodents^54^ and humans^55^. The uncertainty of outcome may trigger a state of stress^56^. Elevated serotonin via the pharmacological inhibition of the 5-HTT and KO (serotonin is elevated in the brain of 5-HTT KO rats^57,58^) promotes movement when coping with stress in rodents^59,60^. KO of 5-HTT may facilitate motor activity when coping with the stress of uncertainty^61^. Besides faster movement, 5-HTT KO rats may also be faster in decision-making as serotonin is speculated to encode reward loss-related prediction error^62,63^. Further, excitation of serotonergic neurons increases the learning rate (as a function of the degree of uncertainty^64^) in a probabilistic choice task^54^. Also, prior research demonstrated that serotonin modulates flexible behavior in changing environments^34,65^. Based on the current work, we cannot dissociate the role of serotonin in non-decision (motor activity) and decision time during outcome generalization. It would be an important topic for future research. In sum, elevated 5-HT might facilitate the adaptation of individuals to changing environments faster. Faster outcome generalization is important to survive in changing environments, but it is not enough to live healthily. In humans, a faster RT to stimuli was observed in both anxious and drug-using individuals^25^. For example, highly anxious individuals were found to process negative information faster at the initial phase of the experiment they were tested in ref. ^66^. Depressed patients also responded faster when processing negative as well as positive information^67^. Also, individuals carrying the short allelic variant of the serotonin transporter-linked polymorphic region showed a faster RT to positive stimuli^68^.

In the initial sessions of stimulus generalization, KO rats responded to the stimuli faster than WT rats. However, as the sessions increased, RT in WT rats gradually decreased while KO rats did not change significantly. There might be a learning effect explaining why KO rats were less able to update the changes in the environment. It is important to note that although the RT had changed, the generalization accuracy, error, bias, and curve stayed similar between KO and WT rats. Furthermore, the generalization curve showed that both KO and WT rats were able to generalize the GSs properly, in which responses to the GSs were more robust when the similarity of the GSs was closer to the CSs. Taking together, WT rats might learn to adapt to changes in environmental stimuli, while KO rats need more training (stimulus exposures) to adapt^35,53^. In our previous experiment^41^, the stage of reduced reward contingencies was not introduced to the animals. It was found that 5-HTT KO rats’ RT gradually decreased as the sessions increased during stimulus generalization, while the RT gradually increased in WT rats. Since GSs were not associated with rewards but are physically similar to CSs, responding to GSs repeatedly may be another source of stress, in which KO rats respond faster than WT rats. Taking the current and previous results into account, 5-HTT knockout in rats might lead to a reduced ability to update information from the novel but similar environmental stimuli.

Generalization is the ability of animals, including humans, to adapt to the environment. Proper generalization is the key to survival. If this basic ability is disturbed, it will lead to inadequate adaptation to environmental changes and can cause brain disorders. Under the biological framework of RDoc, generalization may serve as one of the common dimensions of comorbidity^16^. Animal models displaying comorbidities can be useful to reveal the common biological mechanisms of the comorbidity. For example, 5-HTT knockout affects rodent models of anxiety/depression-like behaviors and modulates drug self-administration^17,30,31^. The current study investigated the pattern of information processing in 5-HTT KO rats when the environment changes in two aspects, the features of the stimulus itself and the outcome predicted by the stimulus. During the outcome generalization, 5-HTT KO rats processed the perceptual information faster than WT rats. During stimulus generalization, KO rats responded to the stimuli faster than WT rats initially. However, as the sessions increased, WT rats appeared to increase their response speed. We conclude that not only stimulus generalization, but also outcome generalization can serve as a basic dimension of disorders, in the RDoC framework, caused by 5-HTT downregulation.

## Supplementary information

Supplementary Methods

Supplementary Figure 1

Supplementary Figure 2
