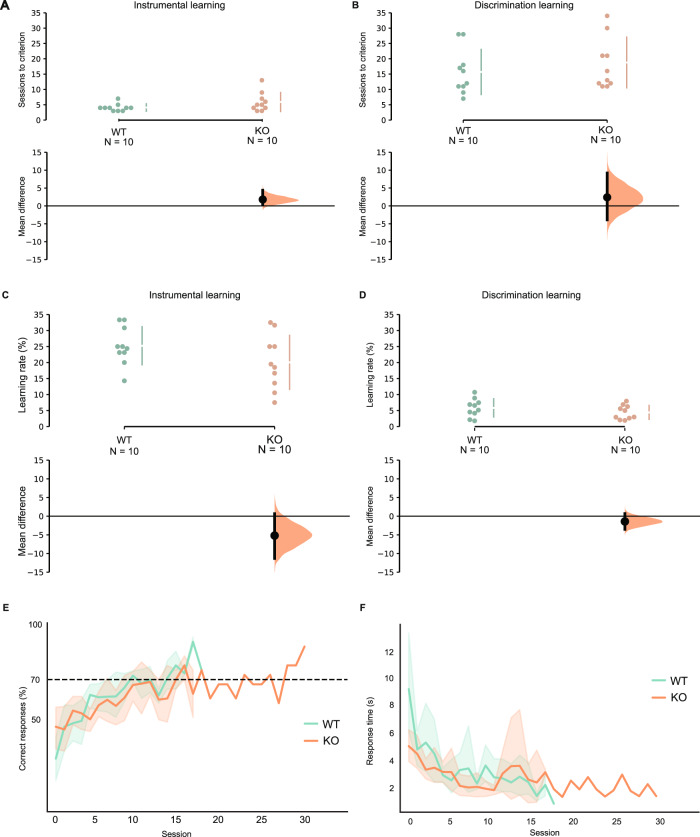# Correction: Knockout serotonin transporter in rats moderates outcome and stimulus generalization

**DOI:** 10.1038/s41398-022-02098-3

**Published:** 2022-08-05

**Authors:** Chao Ciu-Gwok Guo, Tao He, Joanes Grandjean, Judith Homberg

**Affiliations:** 1 Department of Cognitive Neuroscience, Donders Institute for Brain, Cognition, and Behaviour, Radboud University Medical Centre, Nijmegen, The Netherlands; 2 School of Psychological and Cognitive Sciences, Peking University, Beijing, China; 3 Department of Radiology and Nuclear Medicine, Radboud University Medical Centre, Nijmegen, The Netherlands

**Keywords:** Learning and memory, Psychiatric disorders, Predictive markers, Molecular neuroscience

Correction to: *Translational Psychiatry* 10.1038/s41398-020-01162-0, published online 07 January 2021

The original version of this article unfortunately contained an error. The authors state that in the original article they described in the first sub-section of Results (in lines 207–209) that “An estimated Bayes factor from the Bayesian t test (BF_01_ = 1.463) suggested that it was 1.067 times more likely there was no genotype difference of learning rate than there was genotype difference (see Fig. 2C).” The BF_01_ = 1.463 is a mistake. It should be revised to BF_01_ = 1.067 that “An estimated Bayes factor from the Bayesian t test (BF_01_ = 1.067) suggested that it was 1.067 times more likely there was no genotype difference of learning rate than there was genotype difference (see Fig. 2C).” In addition, in the original file of Fig. 2B, the vertical gapped lines for KO group were accidentally placed in the wrong position when aligning the whole figure 2. The authors apologize for the errors. The original article has been corrected.