# Supplementary material for: Knockout serotonin transporter in rats moderates outcome and stimulus generalization
Source: Transl Psychiatry. 2021 Jan 7;11:25. doi: 10.1038/s41398-020-01162-0 (PMC7791109; doi:10.1038/s41398-020-01162-0)
Supplement: Supplementary file 1 — Supplementary Methods [file 41398_2020_1162_MOESM1_ESM.docx]

# Supplementary

### Training and test

### General

Before every training and test day, rats were moved to the testing room from their housing room with their cages covered by a black blanket to avoid light. They stayed in the room for at least thirty minutes for habituation to the novel environment. All rats were handled for 3 days before training in the touchscreen box. Rats were trained and tested every day. The behavioral procedure was modified from a previous touchscreen task developed by us [^1^](https://paperpile.com/c/LsoeuX/Z7AD). A schematic view of a rat in a chamber and trial procedure are presented in Figure 1. To better control the parameters of the stimulus, we used disc-shaped black and white gratings (size: 36cm^2^, contrast: 1.0, special frequency: 2.0, degree of the angles: varied) as the stimulus images in this study. All images/stimuli can be downloaded at <http://hdl.handle.net/11633/aadis56o>. Rats were trained and test stage-by-stage, including pre-training, instrumental conditioning, discrimination training, additional discrimination training with an artificial negative effect, and generalization test.

### Pre-training

To train the rats to obtain rewards by touching the screen, they had to associate the experience of touching an image (a white circular disc, size: 2.88$\pi$cm^2^) on the screen with sucrose pellet delivery. Rats received one session per day during training. Each session consisted of 30 trials with an inter-trial interval (ITI) of 10 seconds. Each trial lasted for 30 seconds. When rats successfully earned the sucrose by touching the image on the screen after seven days of training, they passed through to the next stage of instrumental conditioning.

### Instrumental conditioning

To train the rats that they could only be rewarded if they sequentially touched two pictures at different positions, they were allowed to associate the experience of touching a stimulus image presented on the central window with a subsequent touch on another reporter image (a Chinese character ‘靈’) presented on either the left or the right window. Then they received a sucrose pellet as a reward. The image of ‘靈’ was, because of its complex but the symmetric shape, potentially attractive to rats without bias. Figure 1B presents a representative picture demonstrating reward conditioning. The stimulus consisting of 65° gratings signaling two sucrose pellets was termed the high reward (HR) stimulus, and the stimulus consisting of 25° gratings signaling one sucrose pellet was termed the low reward (LR) stimulus. The two stimulus images, which served as LR stimulus or HR stimulus, were counterbalanced between rats and within each genotype. There were three substages. In the first substage rats were subjected to 30 LR trials; in the second substage rats were subjected to 30 HR trials; in the last substage rats were subjected to 20 LR trials and 20 HR trials. The position of the reporter image was randomized in all three substages, and in the last substage also the order of the trials was random. The ITI ranged from 15 to 30 seconds between trials. Once a rat successfully completed 90% of the trials in one session, it proceeded to the next (sub)stage of training the next day.

### Discrimination training

To train rats to remember that the two stimulus images represent HR and LR values, respectively, they were trained to distinguish between the stimuli of 25° and 65° until reaching a criterion. Different from the previous stage is that, after touching the stimulus image, two reporter images ‘靈’ were subsequently displayed on the left and right windows at the same time (see figure 1C). Rats earned one sucrose pellet by touching ‘靈’ on the left in a trial presenting the LR stimulus. Correspondingly, rats earned two sucrose pellets by touching ‘靈’ on the right in a trial presenting the HR stimulus. If no selection was made within 40 seconds, no sucrose pellet was delivered and the house-light was turned on for 5 seconds. The position of the correct reporter image was counterbalanced across animals and within each genotype. Correction trials were applied when rats made the wrong choice or omitted the prior trial but were not calculated in the total 40 trials. Rats were trained one session per day. Each session consisted of 20 LR trials and 20 HR trials. The order of trials was random and no more than three consecutive trials were of the same type. Once a rat reached 70% correct trials out of the in total 40 trials in a session for three consecutive days, the rat proceeded to additional discrimination training (see Figure 1).

### Additional discrimination training with artificial negative affect

To artificially increase the chance of experiencing the negative affect, 25% of trials with correct responses were not reinforced by rewards. The additional training was identical as described above for discrimination training, except that every fourth correct trial of each trial type was not rewarded with sucrose, resulting in a reduced probability of reinforcement to 75%. In this additional training, the house-light (as a punishment) was not turned on during incorrect or omitted trials to potentially decrease the experience of negative affect from self-mistake. Rats were trained until reaching 70% of trials were made correctly in a session for two consecutive days (see Supplementary Figure 1). Successfully trained rats were tested for generalization (see below).

### Generalization test

Rats received three identical generalization sessions on three separate days. Between each day, rats had to maintain the discrimination accuracy (at least 70%) as same as during the additional discrimination training stage. If the accuracy was lower than 70%, extra training was taken until 70% of trials were executed correctly in a session for two consecutive days. Each session consisted of seventy-two trials across twelve blocks separated by one minute. Each block consisted of six trials presenting five different stimuli. The ITI ranged from 15 seconds to 30 seconds randomly. The order of the trials was a trial presenting a trained stimulus followed by a trial presenting a novel stimulus. The sequence of the trials in each block was counterbalanced across the twelve blocks. As shown in Figure 1D, stimuli consisting of 65° and 25°gratings, the HR and LR stimuli applied during the discrimination stage, were termed “trained stimuli”. The other three novel stimuli were identical to the trained stimuli with an exception that the gratings degree was 5°, 45° or 85°, respectively. The procedure of the trials presenting the novel stimuli was similar to the trials presenting the trained stimuli, but no rewards could be earned after a reporter image was touched by the rats.

## References

1 [Guo CC-G, Verheij MMM, Homberg JR. The serotonin transporter modulates decisional anhedonia. doi:](http://paperpile.com/b/LsoeuX/Z7AD)[10.1101/2020.07.08.190405](http://dx.doi.org/10.1101/2020.07.08.190405)[.](http://paperpile.com/b/LsoeuX/Z7AD)

# Figure legends

**Supplementary Figure 1. Data distributions.** Density estimation of original data (dark blue curve) and the density estimates from 100 generated data sets from the posterior predictive distribution (light blue curves). Observed data and predictive data have similar distributions. (A) RT in the stage of discrimination (B) the ratio of correct responses in the stage of discrimination. (C) RT in the stage of discrimination with artificial negative affect. (D) the ratio of correct responses in the stage of discrimination with artificial negative affect. (E) RT during generalization. (F) the ratio of generalization accuracy. (G) the ratio of generalization error. (H) the ratio of generalization bias. RT: response time.

**Supplementary Figure 2. Response time (RT) during generalization. (A) Genotype effect on RT.** The response time between KO and WT rats across all sessions and stimuli. There was no significant effect of genotype. **(B) Session effect on RT.** The response time across three sessions in all stimuli and genotypes. There was no significant effect of the session. **(C) Stimulus effect on RT.** The response time to each stimulus in all sessions and genotypes. There were significant effects of each stimulus. Note: KO (N=7); WT(N=8). The points between solid lines represent the mean of the group; The dots represent individual data; The hills represent the probability distribution of the individual data; The range of the box represents the interquartile range; The vertical line in the colored box represents the group median; The range of colored box with whiskers on both sides represent the minimum and maximum data range; Data outside the whiskers are outliers denoted by the symbol ⧫.
