# Supplementary figures and images for: Knockout serotonin transporter in rats moderates outcome and stimulus generalization
Source: Transl Psychiatry. 2021 Jan 7;11:25. doi: 10.1038/s41398-020-01162-0 (PMC7791109; doi:10.1038/s41398-020-01162-0)

**A**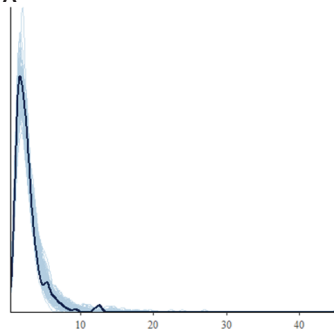**B**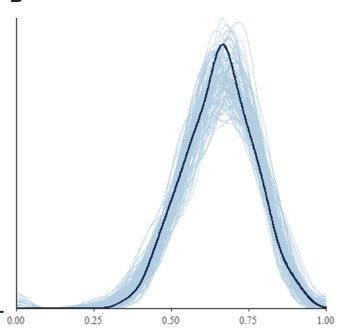**C**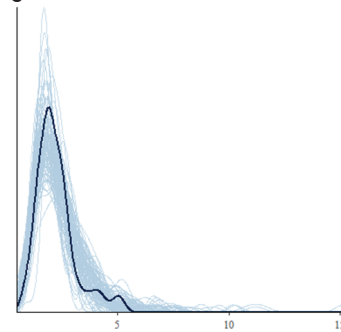**D**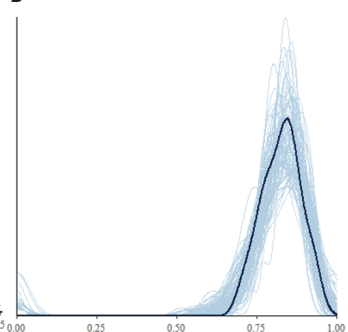**E**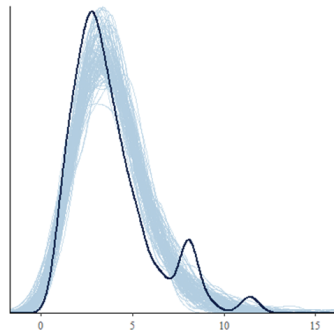**F**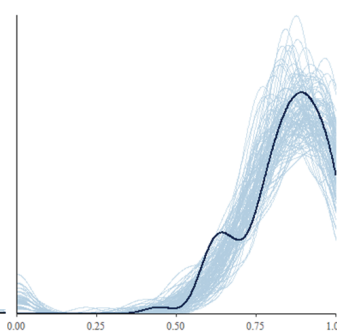**G**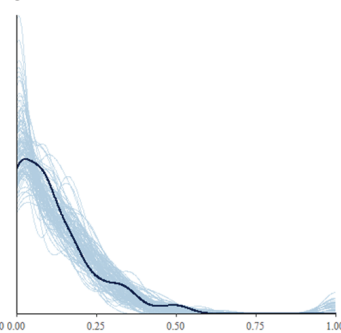**H**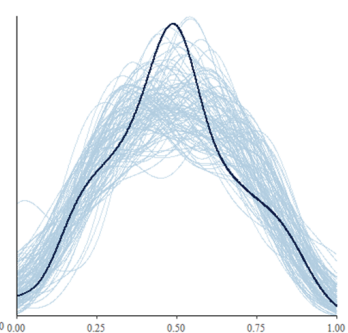

Supplement: Supplementary file 2 — Supplementary Figure 1 [file 41398_2020_1162_MOESM2_ESM.pdf]

**A**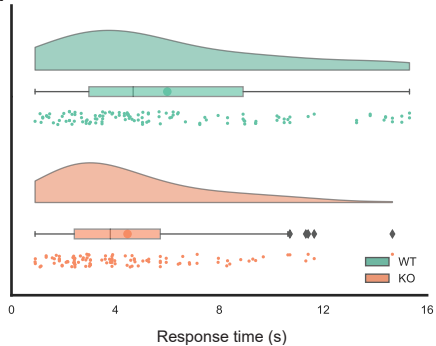**B**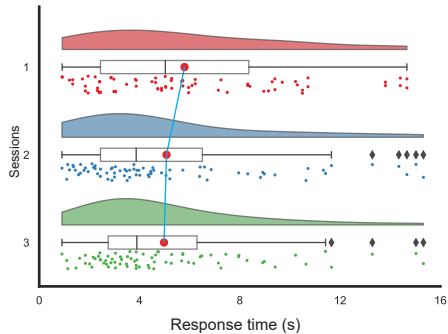**C**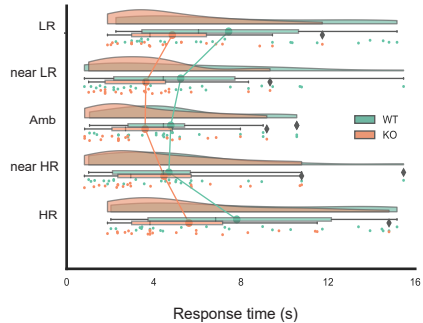

Supplement: Supplementary file 3 — Supplementary Figure 2 [file 41398_2020_1162_MOESM3_ESM.pdf]
